# Comparative performance of ex situ artificial solid electrolyte interphases for Li metal batteries with liquid electrolytes

**DOI:** 10.1016/j.isci.2021.102578

**Published:** 2021-05-21

**Authors:** Francesca Lorandi, Tong Liu, Marco Fantin, Joe Manser, Ahmed Al-Obeidi, Michael Zimmerman, Krzysztof Matyjaszewski, Jay F. Whitacre

**Affiliations:** 1 Department of Chemistry, Carnegie Mellon University, 4400 Fifth Avenue, Pittsburgh, PA 15213, USA; 2 Ionic Materials, Inc., 10-L, Commerce Way, Woburn, MA 01801, USA; 3 Department of Mechanical Engineering, Carnegie Mellon University, 5000 Forbes Avenue, Pittsburgh, PA 15213, USA; 4 Scott Institute for Energy Innovation, Carnegie Mellon University, 5000 Forbes Avenue, Pittsburgh, PA 15213, USA

**Keywords:** electrochemical energy storage, electrochemistry, energy materials, materials chemistry

## Abstract

The design of artificial solid electrolyte interphases (ASEIs) that overcome the traditional instability of Li metal anodes can accelerate the deployment of high-energy Li metal batteries (LMBs). By building the ASEI *ex situ*, its structure and composition is finely tuned to obtain a coating layer that regulates Li electrodeposition, while containing morphology and volumetric changes at the electrode. This review analyzes the structure-performance relationship of several organic, inorganic, and hybrid materials used as ASEIs in academic and industrial research. The electrochemical performance of ASEI-coated electrodes in symmetric and full cells was compared to identify the ASEI and cell designs that enabled to approach practical targets for high-energy LMBs. The comparative performance and the examined relation between ASEI thickness and cell-level specific energy emphasize the necessity of employing testing conditions aligned with practical battery systems.

## Graphical abstract

**Figure:**
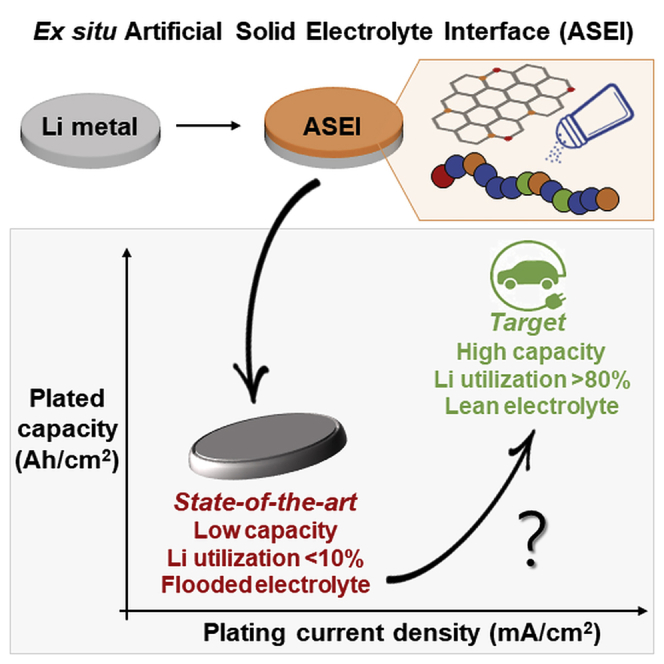

## Introduction

High-energy batteries are key to a low-carbon economy. Electric vehicle (EV) sales are predicted to increase ∼16 times by 2035 (Rietmann et al., 2020), while the declining cost of intermittent renewable electricity is boosting the demand for energy storage (Ziegler et al., 2019). However, commercial Li ion batteries (LIBs) are approaching their specific energy limits. Rechargeable Li metal batteries (LMBs) have the potential to achieve much higher specific energy (Lin et al., 2017; Albertus et al., 2018; Liu et al., 2019).

According to the targets set worldwide, next-generation batteries should reach specific energies of 350–450 Wh/kg at the cell level and cycle life of 1000 deep-discharge cycles (U.S. Department of Energy and Vehicle Technology Office, 2019; Europe Council For Automotive R&D, 2019). LMBs use Li metal as anode, which has theoretical specific capacity of 3860 mAh/g and low reduction potential of −3.04 V (versus the standard hydrogen electrode) (Schmuch et al., 2018). The promise of Li anode triggered even more aggressive targets, such as Battery500's goal of manufacturing LMBs with specific energy exceeding 500 Wh/kg and >1000 cycles lifetime (U.S. Department of Energy, 2020).

**Figure 1:**
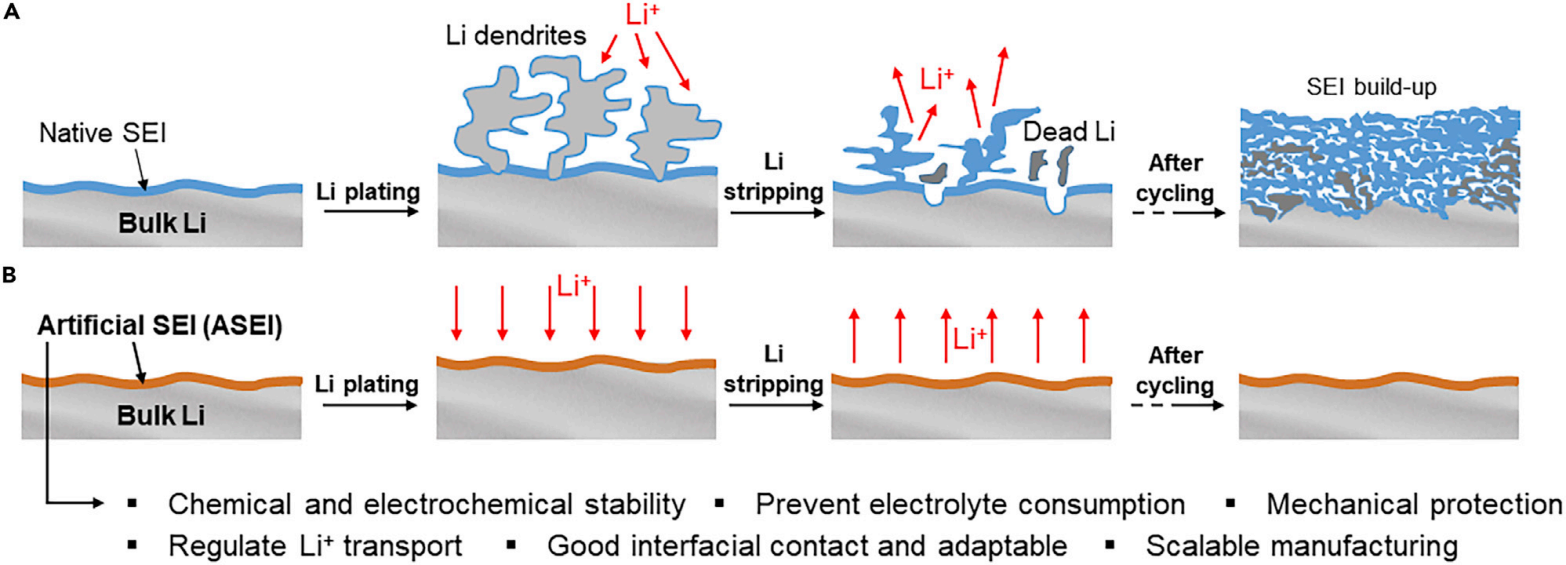
Role of an ASEI on Li metal anode (A) SEI build-up and consequent electrolyte and Li consumption during Li plating/stripping cycles of an LMB with bare Li anode and conventional liquid electrolyte. (B) Uniform Li plating/stripping enabled by coating Li metal with an optimal ASEI.

However, the implementation of Li metal anodes is fundamentally limited by (i) the high reactivity of Li metal and (ii) uneven Li electrodeposition onto itself when charging an LMB (Tikekar et al., 2016b; Zheng et al., 2020; Fang et al., 2019). Conventional aprotic liquid electrolytes react with Li metal forming an ionically conductive but inhomogeneous and fragile solid electrolyte interphase (SEI) (Figure 1A). The uneven SEI morphology creates preferential sites for Li nucleation, driving the formation of Li dendrites or whiskers (Tikekar et al., 2016b; Fang et al., 2019; Gao et al., 2020). These structures can penetrate the separator and ultimately cause an internal short circuit, thus undermining the safety of LMBs. The poor mechanical strength of the SEI results in the formation of cracks that further expose the electrolyte to Li metal, causing gradual electrolyte and Li depletion. The electronically insulating SEI progressively buildup, isolating some metallic Li that during stripping becomes “dead” Li. Therefore, the coulombic efficiency (CE = amount of Li that can be stripped/amount of plated Li) is low (Xiao et al., 2020). Moreover, Li anodes undergo substantial volume changes during cycling, which contribute to deteriorate the interfacial stability (Zheng et al., 2020).

To achieve long-term stable cycling of LMBs, vast research has focused on engineering the Li anode or/and the electrolyte (Cheng et al., 2017; Yu et al., 2020a; Li et al., 2019; Gao et al., 2020). Encouraged by the growing understanding of properties and composition of SEIs on Li metal, several approaches were developed to build either *in situ* (i.e. in an assembled cell) or *ex situ* an artificial SEI (ASEI) (Figure 1B) with ideal features to maximize battery cycle life (Zhang et al., 2018; Xu et al., 2019; Yu et al., 2020c). Particularly, the *ex situ* approach provides unique control and tunability of the ASEI structure and composition. Ideally, ASEI-coated Li metal can be handled in open air, allowing for the manufacture of LMBs in dry room environments that are commonly used for manufacturing LIBs (Tikekar et al., 2016b; Shen et al., 2019; Duffner et al., 2021).

An effective ASEI (i) is chemically and electrochemically stable, (ii) prevents solvents from reacting with Li metal while regulating Li ion transport, (iii) has sufficient mechanical strength to withstand volume changes during cycling and suppress dendrites, (iv) lowers the Li nucleation energy barrier and assists the rapid Li^+^ transport; (v) adapts to the Li metal surface with optimal contact, thus forming a conformal interface; (vi) is manufactured through facile and scalable methods (Zheng et al., 2020; Xu et al., 2019; Yu et al., 2020c). Thus, the design of optimal ASEIs can enable the deployment of LMBs meeting the targets of high specific energy and long cycle life.

This review compares and discusses the cycling performance of a broad variety of *ex situ* ASEIs on Li metal. Representative organic, inorganic, and hybrid organic/inorganic ASEIs have been selected, and their structures, functionalities, and mechanical properties are categorized to help the definition of common features that contribute toward good performance and extended battery cycle life. Typical design principles for effective ASEIs were recently reviewed, (Yu et al., 2020a, 2020c) and the reader is referred to those works for further details on the structures of effective coating materials. In other reviews, the performance of ASEI-coated Li metal anodes was generally discussed in relation to offered improvements in comparison with unprotected Li metal anodes (Xu et al., 2019; Zhou et al., 2020). Herein, the focus is on comparing the performance of cells with ASEI-coated electrodes, in order to identify the requirements in terms of ASEI and cell design to obtain stable LMBs with high specific energy. The performance of ASEI-coated Li metal electrodes and Cu current collectors in symmetric cells and full cells are discussed by using comparative graphs that picture the state-of-the-art in relation to practical targets for high-energy batteries. This review considers the implementation of ASEI-coated Li metal in conjunction with liquid electrolytes, as the latter are currently used for large-scale manufacturing of LIBs, and therefore enabling their use in LMBs can allow for a more rapid deployment of this technology. Finally, both academic and industrial research on ASEI is considered, including patents in the discussion.

### Patent landscape

The number of patents on protective coatings for Li metal anodes has increased over the past 4/5 years, in line with the renewed academic interest in LMBs and the emerging concept of ASEI for stable Li metal (Lin et al., 2017; Tikekar et al., 2016b). The term ASEI is scarcely used in patents, where “coated anode” and “protective coating/film/layer” are more common terms. Herein, we considered a pool of 21 patents, from American, Japanese, and Korean companies, as well as American academic institutions.

Rather than proposing innovative coating materials, the majority of analyzed patents described a method to form a coating on the electrode or to assemble a cell with the inclusion of one or more protective layers for Li metal. Hybrid organic/inorganic coatings are more common in the patent landscape than in the academic literature. This preference for hybrid materials and the tendency to stack multiple layers of various types suggest that combining the advantages of different available materials is a promising and rather simple strategy to obtain an effective ASEI.

## Structure-function relationships for ASEI materials

The materials employed for *ex situ* generated ASEIs were divided according to their nature into organic, inorganic, and hybrid organic/inorganic coatings. Within each category, the ASEIs can be grouped according to common characteristics or functionalities that helped rationalize their performance in battery tests, which are analyzed in the following sections.

### Organic ASEIs

Organic ASEIs include polymers and carbonaceous materials, and they are among the most explored in academic research. Polymer coatings benefit from flexible and highly tunable structures and are generally manufactured through simple solution-processing methods (see “Manufacturing and thickness of ASEIs” section) (Li et al., 2020b; Lopez et al., 2019). The following paragraphs present the main categories of organic ASEIs based on their structural features and corresponding functions.

#### Reactive polymers

**Figure 2:**
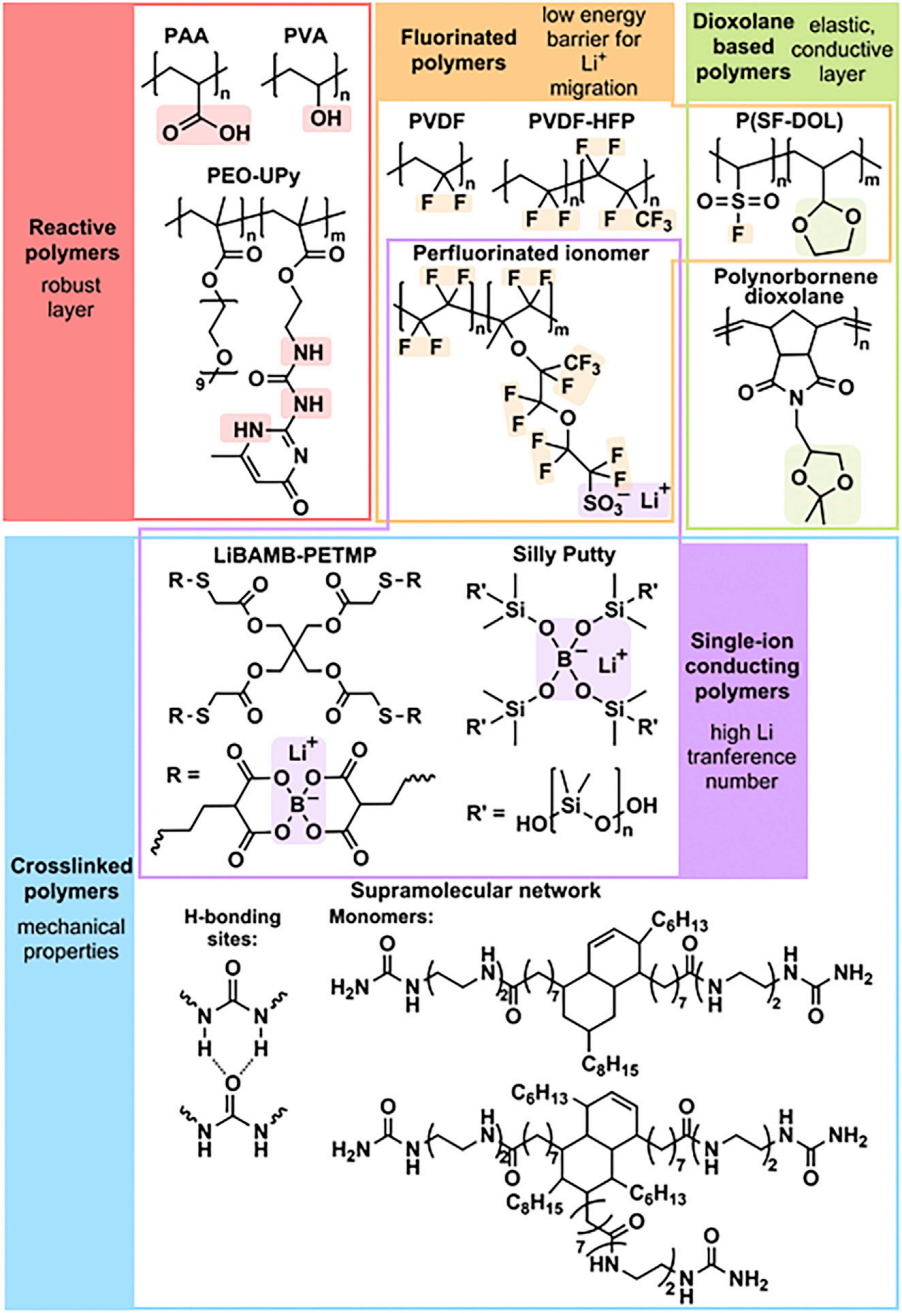
Examples of polymer ASEIs grouped according to their main structural features It should be noted that several polymers pertain to more than one category.

Carboxyl groups, hydroxyl groups, and –NH functionalities in polymer structures react with Li metal forming lithiated analogous (Li et al., 2018a; Zhong et al., 2019; Zhao et al., 2019b). Therefore, polyacrylic acid (PAA) (Figure 2), poly(vinyl alcohol) (PVA) (Figure 2) and polysaccharides were employed to create robust ASEIs on Li metal. In particular, PAA grafted onto the surface of Li metal generating a tough and highly flexible ASEI, owing to the polymer stretchability. In addition, a copolymer of poly(ethylene glycol) methyl ether methacrylate and a methacrylate monomer with pendant ureidopyrimidinone units (PEO-UPy, Figure 2) was used to form a strongly bound layer on Li metal, due to the reactive –NH groups in UPy units (Wang et al., 2020). Moreover, quadrupole-hydrogen-bonding interactions between UPy dimers resulted in self-healing nature of the copolymer, thus ensuring durable performance.

#### Fluorinated polymers

Fluorinated polymer ASEIs have two important properties that favor smooth Li electrodeposition: (i) a low activation barrier for Li^+^ transport and (ii) high interfacial energy between the anode surface and polymer coating, which hinders the growth of dendritic Li structures (Tu et al., 2017; Xu et al., 2018; Luo et al., 2018; Stalin et al., 2020b). The interfacial energy is maximized by the formation of LiF-rich interphases with high surface tension. Perfluorinated polymer ASEIs with low surface energy enhanced the interfacial energy causing the formation of larger Li nucleating sites, and thus more uniform deposition (Stalin et al., 2020b). Consequently, poly(vinylidene fluoride) (PVDF, Figure 2) (Luo et al., 2018; Xiao, 2019a, 2019b), polytetrafluoroethylene (PTFE) (Xiao, 2019a, 2019b), and PVDF-*co*-hexafluoropropylene (PVDF-HFP, Figure 2) (Xu et al., 2018) were extensively studied as anode coating in LMBs.

#### Porous polymers

Dendrites have micrometric size; therefore nanoporous polymer membranes suppress their growth. PVDF-HFP combines the advantages of fluorinated coatings to a nanoporous structure that could prevent dendrites (Xu et al., 2018). Other porous polymers used as ASEIs include covalent organic frameworks (Chen et al., 2020) and polymers of intrinsic microporosity (PIMs) (Moon et al., 2019), which are glassy polymers presenting spirocenters that create high-tortuosity channels. The nanopores in these materials are inaccessible to bulky anions, and even to solvent molecules in the case of a PIM with pore size < 1 nm. Thus, these nanoporous membranes allowed exclusive and uniform transit of Li ions. In alternative, acid treatment can be used to create nanoporosity in polymer membranes (Zhu et al., 2017).

#### Dioxolane-based polymers

1,3-Dioxolane (DOL) is a common component of ether-based liquid electrolytes, responsible for the formation of elastic and ionically conductive oligomeric species (polyDOL) in the SEI. As a result, typical ether-based electrolytes form a more stable SEI compared to carbonate-based electrolytes (Tikekar et al., 2016b). PolyDOL-rich layers are commonly prepared on Li metal *in situ,* by electrochemical pretreatment of Li electrodes in a DOL-rich electrolyte (Kozen et al., 2017; Ma et al., 2017). However, they could also be formed *ex situ* by using DOL-based polymers, including a polynorbornene derivative (Gao et al., 2017), and a copolymer of vinyl sulfonyl fluoride and 2-vinyl-DOL (P(SF-DOL)) that formed an interphase simultaneously rich in polyDOL and LiF (Figure 2) (Gao et al., 2019).

#### Cross-linked polymers

Polymer networks generally exhibit superior mechanical strength to linear polymers, and their nanometer-sized meshes can prevent the growth of dendrites. Networks based on poly(ethylene oxide) (PEO) and fluorinated polymers have been employed as ASEIs, due to the conductivity of PEO and the benefits of F-rich interphases (Stalin et al., 2020b). Moreover, dynamic polymer networks bearing reversible cross-links can exhibit interesting rheological properties and self-healing ability. For example, Silly Putty (Figure 2) is a commercial toy composed of a polydimethylsiloxane backbone and dynamic boronic esters linkages (Liu et al., 2017a). Its viscoelastic nature made Silly Putty an ideal ASEI material that responded to localized increases in Li growth by stiffening and suppressing the protrusion, and subsequently recovered a liquid-like nature. Similarly, a supramolecular ASEI constituted by oleic acids, multidentate amines, and urea (Figure 2) displayed high elasticity, solid-liquid nature and self-healing ability, limiting the formation of pinholes and inhomogeneities during cycling (Zheng et al., 2016).

#### Single-ion conducting (SIC) polymers

SIC polymers possess high Li transference number, thus enabling highly efficient Li^+^ transport (Tikekar et al., 2016b; Zhang et al., 2017a; Li et al., 2018b). The use of an ASEI with single-ion transport ability can raise the transference number of the entire bulk liquid electrolyte, with negligible effect on its conductivity (Tu et al., 2017). SIC polymer ASEIs can be composed of polymers bearing anionic sites in their backbone, such as the Nafion-type perfluorinated ionomer LITHion^TM^ (Tu et al., 2017), or by covalently linking anionic units to polymer chains, as in a mechanically strong network possessing SIC bis(allylmalonato) borate (BAMB) units (Figure 2) (Deng et al., 2019).

#### Polymers containing ethylene oxide or cyano units

PEO and polyacrylonitrile (PAN) are extensively studied as battery electrolytes due to their ability to conduct Li ions (Ma et al., 2014a; Lang et al., 2017; Zhang et al., 2017b; Wang et al., 2019). In PAN-based ASEIs, polar CN groups with good affinity for Li metal and Li^+^ contributed to protect the electrode and create pathways for smooth ion transport. A similar effect was achieved using another –CN containing polymer, poly(ethyl cyanoacrylate), that in the presence of LiNO_3_ formed a highly conductive and robust ASEI, with a high Young’s modulus of 25 GPa (Hu et al., 2017).

#### Carbonaceous materials

Carbonaceous materials employed as ASEIs include carbon paper, and graphene oxide (GO) and its derivatives (Bai et al., 2018; Kim et al., 2018; Chen et al., 2019c; Gao et al., 2019; Zhao et al., 2018). The vast use of GO and reduced GO (rGO) is motivated by their low cost, high specific surface area and electronic conductivity, and ability to facilitate Li^+^ diffusion on surfaces and within interlayer gaps. Their high mechanical strength allows for withstanding electrode volumetric changes during cycling. Moreover, rGO has high lithiophilicity due to the abundance of functional groups (Kim et al., 2018). The morphology of GO and the presence of dopants can affect the ASEI performance. In particular, macroporous GO offered low tortuosity pathways for enhanced Li^+^ diffusion (Chen et al., 2019c), while phosphorous functionalized rGO (PrGO) showed relatively low overpotential for Li nucleation, because of the strong interactions between P and Li (Kim et al., 2018).

In summary, organic ASEIs generally contain functional groups that (i) react with Li metal; (ii) decrease the energy barrier for Li^+^ transport, and enhance the conductivity; (iii) are anionically charged, to form single Li ions conducting coatings. The mechanical strength of organic ASEIs is increased by forming cross-linked materials, or by using GO and its derivatives. Moreover, dynamic cross-links can provide adaptability and self-healing ability to the ASEI. Finally, nanoporous materials can prevent electrolyte penetration and dendrite growth.

### Inorganic ASEIs

Inorganic ASEIs for LMBs generally showed higher ionic conductivity than organic coatings, as well as higher mechanical strength to contain volume changes at the electrode, and favorable surface energy to regulate the Li^+^ electrodeposition. However, inorganic coatings typically required costly manufacturing methods and their brittleness could prevent long-term performance.

#### LiF-based coatings

Calculations showed that a LiF film has low energy barrier to Li^+^ diffusion, and high and uniform surface energy to minimize charge gradients (Fan et al., 2017). Moreover, an LiF coating could modulate the electrical field next to the electrode, limiting electrolyte decomposition and diminishing the stress on Li metal. In addition, LiF has high mechanical strength with Young’s modulus varying in the range 50-140 GPa. Different manufacturing processes were explored to create a LiF film, including solution-based processes (Lang et al., 2019; Peng et al., 2019; Yuan et al., 2019) and chemical vapor deposition techniques (Chen, 2019; Gopalakrishnannair and Herle, 2017). In addition, metal fluoride salts were coated on Li metal to generate LiF-rich interphases. For example, CuF_2_ formed a polycrystalline ASEI composed of LiF and Cu, exhibiting mixed ionic and electronic conductivity (Yan et al., 2018). On the other hand, SnF_2_ generated a Li-Sn alloy that decreased the barrier to Li^+^ diffusion, and a LiF-rich interphase with enhanced wettability, high surface energy and Young’s modulus of 55.6 GPa (Pathak et al., 2020).

#### Li_3_N-based coatings

Lithium nitrate has high room temperature conductivity (∼6 × 10^-3^ S/cm) and high mechanical strength (Young’s modulus of 46-83 GPa) (Alpen et al., 1977). Therefore Li_3_N films were generated on Li metal, as highly conductive and robust ASEIs (Wu et al., 2011; Ma et al., 2014b; Chen et al., 2019a). In particular, α-phase Li_3_N decreased the energy barrier for Li^+^ migration to a greater extent than β-phase Li_3_N (Chen et al., 2019a).

#### Phosphorous (P)-based coatings

Similarly to LiF, Li_3_PO_4_ exhibits relatively low ionic conductivity (∼10^-8^ S/cm at r.t.) and low electronic conductivity, however it promotes homogeneous distribution of charge density, thus uniform Li electrodeposition (Lehfeldt, 1933; Li et al., 2016; Wang et al., 2017). Moreover, Li_3_PO_4_ coating films had high mechanical strength. Additionally, the amorphous solid-state electrolyte Lithium phosphorous oxynitride (LiPON) was studied as ASEI, owing to its high ionic conductivity and chemical stability against Li metal (Kozen et al., 2017; Liu et al., 2018).

#### Aluminum oxide

The plating of Li^+^ on Al_2_O_3_ films formed covalent Li-Al-O bonds, thus generating a stable and uniform interfacial layer with decreased impedance. Its high ionic conductivity resulted in a decreased energy barrier to Li^+^ migration and enhanced diffusion rate of Li ions (Kozen et al., 2015; Kazyak et al., 2015; Wang et al., 2018). Thus, Al_2_O_3_ acted as a “surfactant” that facilitated uniform Li nucleation and induced a smooth layer-by-layer growth of plated Li. In Li-S batteries, Al_2_O_3_ layers prevented the self-discharge of S and deposition of polysulfides on the anode (Kozen et al., 2015).

#### Others

The formation of Li-rich alloys on Li metal decreased the interfacial resistance, enhancing the rate of Li^+^ transport. Therefore, Sn (Archer et al., 2018) and various metal halide salts including SnF_2_(Pathak et al., 2020) and several metal chlorides (MCl_x_, with M = In, Zn, Bi, As, Ge) (Liang et al., 2017; Liao et al., 2018) were exploited to form alloy-based ASEIs. Other inorganic materials employed as ASEIs include a highly conductive (10^-4^ S/cm) film of Li_3_OCl able to self-regenerate after the formation of cracks (Han et al., 2020), and lithium terminated sulfonated TiO_2_ NPs, whereby the charged polar sulfonated groups repelled polysulfides in Li-S batteries (Kim et al., 2017). Moreover, two dimensional (2D) materials, such as MoS_2_ and MoSe_2_, were employed to form films on Li metal promoting facile intercalation of Li ions and decreasing the interfacial resistance (Choi and Cha, 2020).

Overall, inorganic ASEIs predominantly include metal salts and metal oxides with high mechanical strength and ability to form highly uniform coatings that homogenize the Li^+^ flux. Moreover, several metal salts are capable of alloying with Li, promoting fast Li diffusion and reducing the interfacial resistance.

### Hybrid organic/inorganic ASEIs

Hybrid organic/inorganic ASEIs combine the advantages of both class of materials, displaying high conductivity and mechanical strength typical of inorganic coatings, while overcoming their traditional brittleness, and achieving flexibility and ease of manufacturing typical of organic coatings. It should be noticed that spontaneously formed SEIs by decomposition of the electrolyte and eventual additives generally contain both organic and inorganic components, which can have different roles in stabilizing the device performance (Zhang et al., 2018; Yu et al., 2020c). The design of hybrid ASEIs can highly benefit from advanced synthetic strategies such as controlled radical polymerizations (Corrigan et al., 2020), which enable to graft polymer chains onto or from inorganic particles, obtaining materials with improved uniformity and tunable properties (Li et al., 2020a, 2020b).

#### Metal-based hybrid coatings

Metal and metal oxide nanoparticles were mixed with GO, rGO or doped-GO, to promote homogeneous distribution of NPs that facilitated the nucleation of Li, and to create pathways for uniform Li^+^ transport (Pu et al., 2018; Cho et al., 2019). Polymers were also combined with metal and metal oxide NPs, and metal organic frameworks (MOFs) to impart flexibility to the resulting films, which exhibited higher mechanical strength (Young's moduli in the GPa range) compared to pure polymer films, and thus improved regulation of volume changes (Li et al., 2020a; Liu et al., 2017c; Fan et al., 2020). To achieve uniform distribution of inorganic NPs, preventing aggregation and consequent deterioration of mechanical properties and performance, PAN chains were grafted from yttria-stabilized zirconia (YSZ) NPs by surface initiated atom transfer radical polymerization (SI-ATRP) (Li et al., 2020a; Ribelli et al., 2019). The oxygen vacancies on the surface of uniformly distributed YSZ NPs facilitated the Li^+^ transport.

#### Si-based hybrid coatings

Si and SiO_2_ NPs were employed to form ASEI by either embedding them in polymer matrices to improve the flexibility of the coatings or by employing core-shell structures with polymer chains grafted onto the NPs to form highly uniform films (Yu et al., 2020b; Liu et al., 2017b). In particular, core-shell nanospheres composed of poly(methyl methacrylate) (PMMA) chains grafted from SiO_2_ NPs, with inorganic content >55%, formed a mechanically strong ASEI with triangular nanopores, capable of preventing the growth of dendrites (Liu et al., 2017b).

#### P-based hybrid coatings

Hybrid ASEIs were designed to improve the mechanical properties of highly conductive coatings based on P-containing inorganic materials (Kozen et al., 2017; Zhao et al., 2019a). At the same time, the inorganic phase could reinforce the polymer phase, such as by introducing LiPON in a polyDOL-rich interphase formed by electrochemical reduction of DOL (Kozen et al., 2017).

## Performance in Li|Li symmetric cells and Li|Cu cells

### Selected parameters and targets

Comparative electrochemical performance of ASEI-protected Li metal electrodes are reported herein for symmetric Li|Li cells and Li|Cu cells, whereas the next section discusses full cells composed of various cathode materials coupled with ASEI-coated Li metal anodes. While each ASEI-coated electrode enabled better performance than a bare electrode under identical conditions, the purpose of these sections is to compare and discuss the performance of ASEI-coated electrodes in relation to the coating materials and testing conditions. Since lab-scale tests are typically performed in coin-cells, testing conditions should be aligned to practical pouch-cell conditions to obtain meaningful performance and accelerate the technology transfer (Chen et al., 2019b; Liu et al., 2019). This is important not only for full cell tests but also for symmetric cells, which are commonly used to evaluate the properties of ASEIs, as they allow to separate events occurring at the cathode from the evaluation of coated Li anode.

The performance evaluation takes into account 4 parameters, selected in accordance with a report by Albertus et al. that broadly described the state-of-the-art of LMBs in early 2018 (Albertus et al., 2018). The parameters are: (i) plating current density; (ii) per-cycle plated capacity; (iii) cumulative capacity plated prior to cell shorting, obtained as the per-cycle plated capacity multiplied by the number of cycles; (iv) the fraction of initial Li metal that passed per cycle (i.e. the Li metal effectively utilized during cycling). The performance targets proposed by Albertus et al. are also adopted herein and include (i) a goal of the ARPA-E IONICS program of the US Department of Energy (denoted as “T1”) that targets stable cycling for 500 cycles at a current density and per-cycle capacity of at least 3 mA/cm^2^ and 3 mAh/cm^2^, respectively, with a depth of discharge exceeding 80%; (ii) a cell with a loading of 5 mAh/cm^2^, capable of fast charging at 2C and achieving a cycle life of 2000 cycles at a depth of discharge >80% (“T2”). Bubble graphs were employed to clearly represent and compare multiple parameters in a single graph.

**Figure 3:**
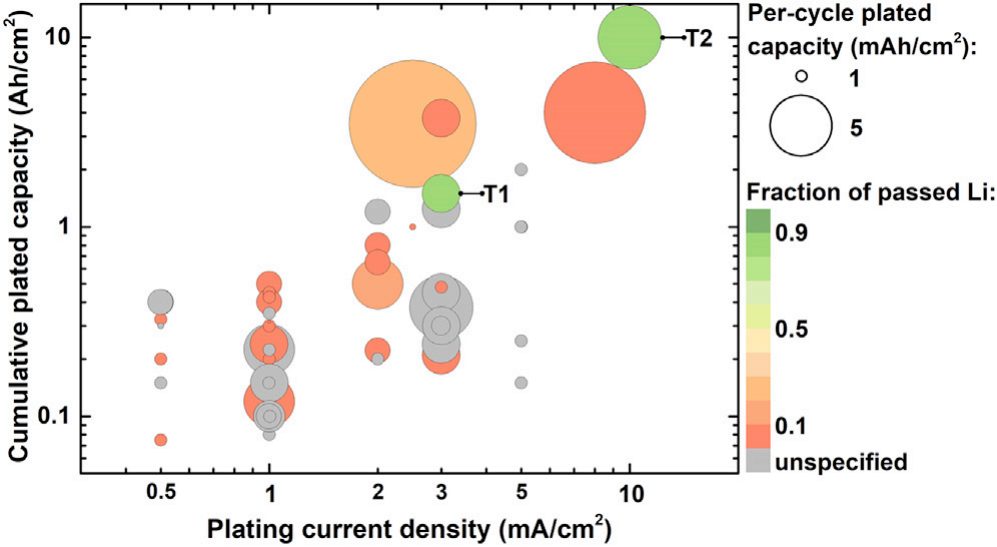
Performance of ASEI-coated electrodes in Li|Li and Li|Cu cells Performance of ASEI-coated Li metal electrodes in Li|Li symmetric cells and ASEI-coated Cu current collectors in Li|Cu cells, analyzed according to 4 parameters: (i) cumulative plated capacity prior to cell shorting or for the reported cycling duration (y axis); (ii) plating current density (x axis); (iii) per-cycle plated capacity (bubble size); (iv) fraction of initial Li metal passed per cycle (bubble color; gray bubbles correspond to lack of information on the thickness of Li foils). The ARPA-E IONICS goal (T1) and the fast-charging goal (T2) were included as reference targets. Details on materials and parameters calculations are available Table S1.

Figure 3 shows the performance of several ASEI-coated Li metal electrodes in symmetric cells and ASEI-coated Cu current collectors in Li|Cu cells. Details on the publications included in the graph are available in the supplemental information. It should be noticed that in several reports the cycling was interrupted before cell failure, when the overpotential was still low and stable. Therefore, the corresponding cumulative plated capacity is likely underestimated. Moreover, the thickness of Li metal electrodes was often omitted in the analyzed works, precluding the calculation of the Li utilization (gray bubbles).

The nature of liquid electrolytes, presence of additives and electrolyte amount were not considered in the performance comparison. Generally, carbonate-based solvents with LiPF_6_ or ether-based solvents with LiTFSI were employed. Carbonate-based electrolytes typically comprised mixtures of ethylene carbonate with either diethyl carbonate, dimethyl carbonate, or ethyl methyl carbonate. Additives such as vinylene carbonate, fluoroethylene carbonate and lithium bis(oxalato)borate were frequently employed. When the electrolyte is in contact with Li metal, these additives contribute to form better SEIs with lower interfacial impedance (Zhang et al., 2018). Ether-based electrolytes generally contained 1,2-dimethoxyethane, DOL, and LiNO_3_ as additives. Ether-based electrolytes form an SEI with relatively high fraction of organic, oligomeric, and elastic components, which provide better stability in comparison to typical SEI formed by carbonate-based electrolytes (Tikekar et al., 2016b). However, the electrochemical stability window of carbonate electrolytes is wider, and therefore they are more suitable for high-voltage operation. While the presence of an ASEI diminishes the electrolyte decomposition by hindering contact with Li metal, electrolyte additives can affect the long-term performance by further improving the stability of the anode/electrolyte interface. Ultimately, the optimization of both liquid electrolyte and ASEI composition will be needed to achieve stable cycling and improved safety in commercial devices. While the electrolyte loading was evaluated only in relation to full-cell performance (see next section), it should be noted that Li|Li and Cu|Li cell tests are often performed with electrolyte flooded cells to prevent eventual failure due to electrolyte consumption. However, one of the role of an ASEI is to minimize the electrolyte consumption, thus enabling to decrease the electrolyte loading.

### Requirements to meet the targets

The bubble graph in Figure 3 encouragingly shows that several works reported performance approaching the targets, in particular the ARPA-E IONICS goal. However, two important issues emerge. First, the majority of reports performed cycling tests under current densities and/or per-cycle capacities that are inadequate to reach target performance. Current densities of ≥3 mA/cm^2^ are recommended to demonstrate that coated electrodes are suitable for practical applications. Second, the fraction of Li passed per cycle is usually <10% (often <1%) of the total Li initially present in the cell, in stark contrast with the targets (>80%). The low Li utilization results from the use of thick Li metal foils (with thickness >150 μm, typically 450–750 μm). The presence of high excess of Li is not only far from practical requirements but also renders reported performance questionable. When using thick electrodes, eventual “soft shorts” (i.e. stable electronic connection between the electrodes) cannot be easily detected, and long-term stable cycling is seemingly observed (Albertus et al., 2018; Xiao et al., 2020; Zhu et al., 2020). Electrochemical impedance spectroscopy measurements are sometimes reported to exclude the occurrence of soft shorts. Nevertheless, limiting the excess Li can simultaneously help detect soft shorts and obtain more meaningful performance. Therefore, it is strongly recommended to employ Li metal foil with a thickness of ≤30 μm, which are commercially available.

An alternative to the use of thin Li foils is the pre-deposition of a fixed amount of Li on Cu current collector, which enables the formation of a Li anode with desired thickness (1 mAh/cm^2^ deposits ∼5 μm of Li). By using this approach, the ASEI can be constructed on the Cu current collector before Li deposition. This can facilitate the manufacturing of the ASEI, as Cu foils can be handled in open air. Upon pre-deposition of a certain amount of Li in a dedicated cell, the device is disassembled and the ASEI-protected thin Li electrode is coupled to another Li foil or to a cathode material for further tests (Zhu et al., 2020). Alternatively, the ASEI-coated Cu current collector can be directly used in Cu|Li cells or as negative electrode in “anode-free” full cells (Nanda et al., 2020). Cu|Li cells are commonly used to measure the CE and sometimes to evaluate the effect of the ASEI on long-term performance. However, in this type of cells the thickness of the Li foil (counter electrode) determines the Li inventory (Xiao et al., 2020). Therefore, thin Li foils should be used also in Cu|Li cells to increase the Li utilization, mimicking practical conditions.

Growing awareness over the need of eliminating excess Li is manifested by the use of thin Li metal foils in recent works. The PEO-UPy self-healing and reactive polymer coating (Figure 2) was applied to 50 μm thick Li metal foils and tested in Li|Li symmetric cells showing stable cycling at a current density of 2 mA/cm^2^ and areal capacity of 4 mAh/cm^2^ for over 125 cycles, with Li utilization of ∼20% (Wang et al., 2020). In a patent application filed by GM Global Technology Operations LLC, consecutive PVDF and PTFE layers were coated on 90 μm Li foils by RF magnetron sputtering (Xiao, 2019b). The coated foils were cycled at a high areal capacity of 10 mAh/cm^2^, obtaining low and stable overpotential for over 350 cycles with an Li utilization of ∼27%.

### Performance of different coating materials

**Figure 4:**
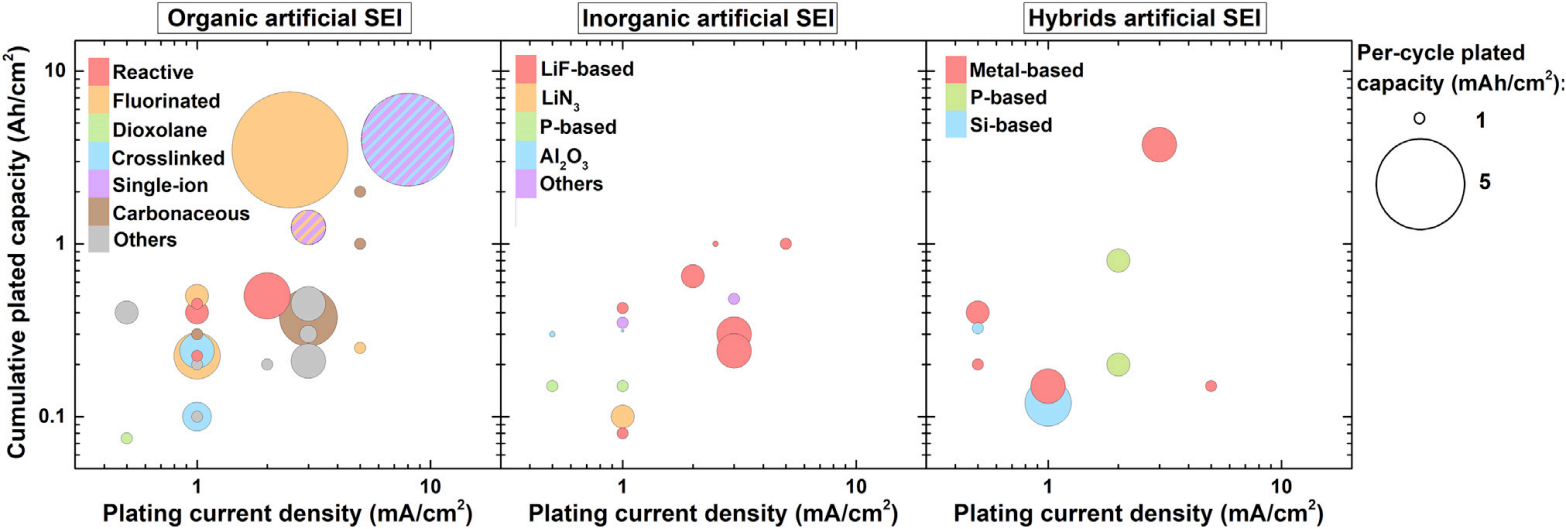
Performance in Li|Li and Li|Cu cells grouped according to the ASEI nature Polymer coatings in the organic ASEI graph are color-coded according to Figure 2.

In order to better analyze the performance of each class of coating materials, the graph in Figure 3 was split in 3 different charts for organic, inorganic, and hybrid ASEIs (Figure 4). The bubbles colors in Figure 4 correspond to the different structures/functionalities of the ASEIs, as described in the previous section. Figure 4 shows that organic ASEI is the category with most examples demonstrating good cycling stability under current densities approaching practical values (≥3 mA/cm^2^), followed by inorganic ASEIs (mainly LiF-based) and a few hybrid ASEIs.

Among organic coatings, very promising performance was achieved by using SIC polymers, in particular the LITHion^TM^ perfluorinated ionomer and the network containing negatively charged BAMB units (Figure 2) (Tu et al., 2017; Deng et al., 2019). Both ASEIs presented Li-coordinating anionic sites attached to a polymer backbone. Notably, both ASEIs also presented a distinct secondary feature, either a fluorinated scaffold or a crosslinked morphology. This observation suggests that, ultimately, a successful ASEI could rely on a combination of different features to ensure prolonged stability of Li metal under practical conditions. The SIC perfluorinated ionomer combined favorable surface energy, a high Li transference number of 0.87, and low activation barrier to Li^+^ migration, typical of fluorinated coatings (Tu et al., 2017). Low and stable overpotential was measured for over 400 cycles at 3 mA/cm^2^ and 3 mAh/cm^2^, in a carbonate electrolyte and in the presence of a nanoporous Al_2_O_3_ separator. On the other hand, in the SIC polymer network, both the crosslinked structure and the efficient transport of Li^+^ contributed to suppress the growth of dendrites (Deng et al., 2019). The relatively weak electrostatic interaction between B^−^ and Li^+^ resulted in high conductivity (3.3 × 10^−5^ S/cm at 25 °C) and high transference number (0.91). Stable cycling was obtained for >500 cycles at a high current density of 8 mA/cm^2^ and per-cycle areal capacity of 8 mAh/cm^2^.

These examples support the notion that the immobilization of anions in “soft” polymer membranes promotes stable Li electrodeposition (Tikekar et al., 2016a; Zhao et al., 2020). This appears in contrast to Monroe and Newman's observation that a stable lithium/polymer interface is achieved when the shear modulus of the polymer is about twice that of lithium (i.e. Young's modulus >6 GPa) (Monroe and Newman, 2005). They further proposed that the introduction of inorganic nanoparticles diminishes polymers' compressibility, enhancing the interface stability, which is necessary to achieve stable Li electrodeposition and suppress deformation forces and dendrite growth. However, these statements are challenged by reported evidences of polymer-based electrolytes with relatively low modulus (typically in the MPa range) enabling long-term stable cycling of Li metal electrodes (Lopez et al., 2019; Zhao et al., 2020; Stalin et al., 2020a). Moreover, dendrite penetration through high shear modulus ceramic electrolytes has been observed and explained considering their electronic conductivity, poor wettability, presence of voids and grain boundaries, and induced local variation in the chemical potential of Li (Ahmad and Viswanathan, 2017; Liu et al., 2020; Porz et al., 2017; Krauskopf et al., 2020). Recent studies suggest that stable Li deposition can be achieved through solid electrolytes and interphases displaying relatively low rigidity but high surface tension and transference number (Lopez et al., 2019; Zhao et al., 2020). SIC units reduce the electric field on Li metal, thus mitigating interfacial instabilities, balancing the insufficient mechanical strength to block the dendrite growth (Tikekar et al., 2016a). The inclusion of SIC units in nanostructured materials such as crosslinked polymers help confine the electrodeposits, while simultaneously improving the mechanical properties (Deng et al., 2019; Zhao et al., 2020). In addition, single-ion conductors can work in tandem with fluorinated coatings to control the morphology of Li deposits, as in the case of the perfluorinated ionomer film (Zheng et al., 2020; Tu et al., 2017).

The beneficial effect of fluorinated polymers is further supported by the ability of a double-layer PVDF-PTFE ASEI to enable stable cycling at 2.5 mA/cm^2^ and 10 mAh/cm^2^ for 350 cycles and relatively high Li utilization, in a concentrated ether electrolyte (Xiao, 2019b). The PEO-UPy polymer is another example of a soft coating layer that provided good cycling stability at a current density of 2 mA/cm^2^ and Li utilization of ∼20% (Wang et al., 2020). The copolymer exhibited a transition from solid-like to liquid-like behavior at high shear strain (i.e. shear-thinning nature). This behavior resulted in a conformal, adaptable and self-healing coating that effectively blocked the proliferation of dendrites. The promising performance of these polymer ASEIs suggests that careful engineering of polymer surface tension and rheology by introducing appropriate functionalities is an effective strategy toward stable Li metal anodes.

The most common approach to improve the performance of polymer coatings is the inclusion of inorganic components in polymer matrices. However, simple blends of inorganics and polymers tend to result in aggregation of inorganic components, which can deteriorate the mechanical properties (Liu et al., 2017b; Nematdoust et al., 2020). Therefore, limited inorganic loadings are generally necessary to preserve the material uniformity. Conversely, inorganic nanoparticles grafted with polymer chains allow for obtaining homogeneous hybrid materials with relatively high loading of inorganic particles, which have been employed as robust and uniform coatings for Li metal (Liu et al., 2017b; Li et al., 2020a). PAN chains grafted from YSZ nanoparticles (PAN-*g*-YSZ) via SI-ATRP formed uniform films on Li metal foils with YSZ content between 13 wt% and 72 wt%. Symmetric cells with PAN-*g*-YSZ (24.6 wt% YSZ NPs) ASEIs on Li metal foils achieved stable cycling for over 2500 hr at 3 mA/cm^2^ and 3 mAh/cm^2^ (Li et al., 2020a). In contrast, a PAN/YSZ blend with similar composition showed much higher overpotential and rapid cell failure, due to aggregation of NPs and inhomogeneity of the ASEI.

**Figure 5:**
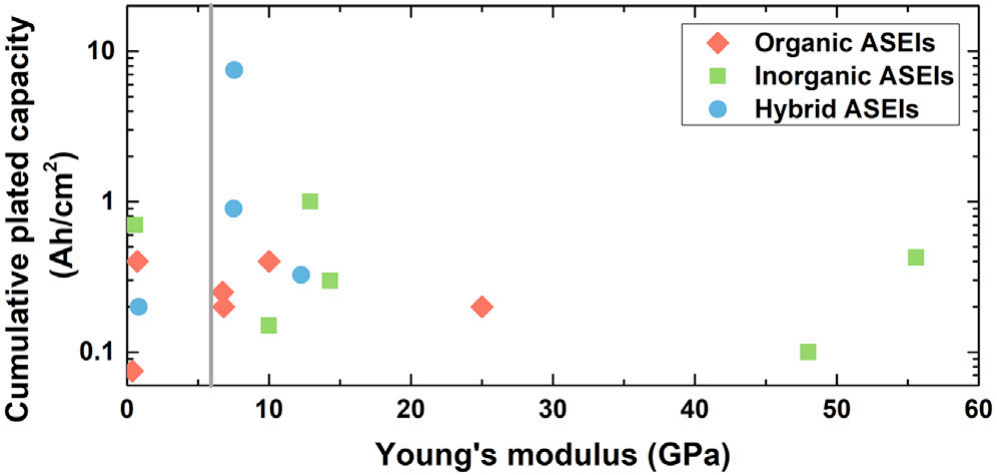
Relation between ASEI Young's modulus and cycling performance Values of Young's modulus of protective films for Li metal analyzed in this report plotted in relation to the cumulative plated capacity realized in symmetric cells using coated Li metal as electrodes. The solid gray line represents the minimum modulus value required to block dendrites according to Monroe and Newman's calculation (~6 GPa). More details are provided in Table S1.

To further evaluate the role of the mechanical strength of an ASEI, Figure 5 shows the cumulative plated capacity achieved by coated Li metal electrodes in symmetric cell tests as a function of the Young's modulus of the ASEI (when reported). Despite having moduli close to or lower than 6 GPa, several organic and hybrid ASEIs enabled long-term stable cycling and relatively high plated capacities, thus challenging Monroe and Newman's predictions. Besides the modulus, other structural features of an ASEI have important contribution in determining optimal performance, similarly to the case of solid-state electrolytes (Zhao et al., 2020; Kong et al., 2020). Therefore, relatively soft materials possessing nanoporous structures, and/or high Li transference number, and/or viscoelastic nature could still provide sufficient protection for long-term stable LMBs.

## Performance in Li metal batteries

### Selected parameters, targets and considerations on cathode materials

**Figure 6:**
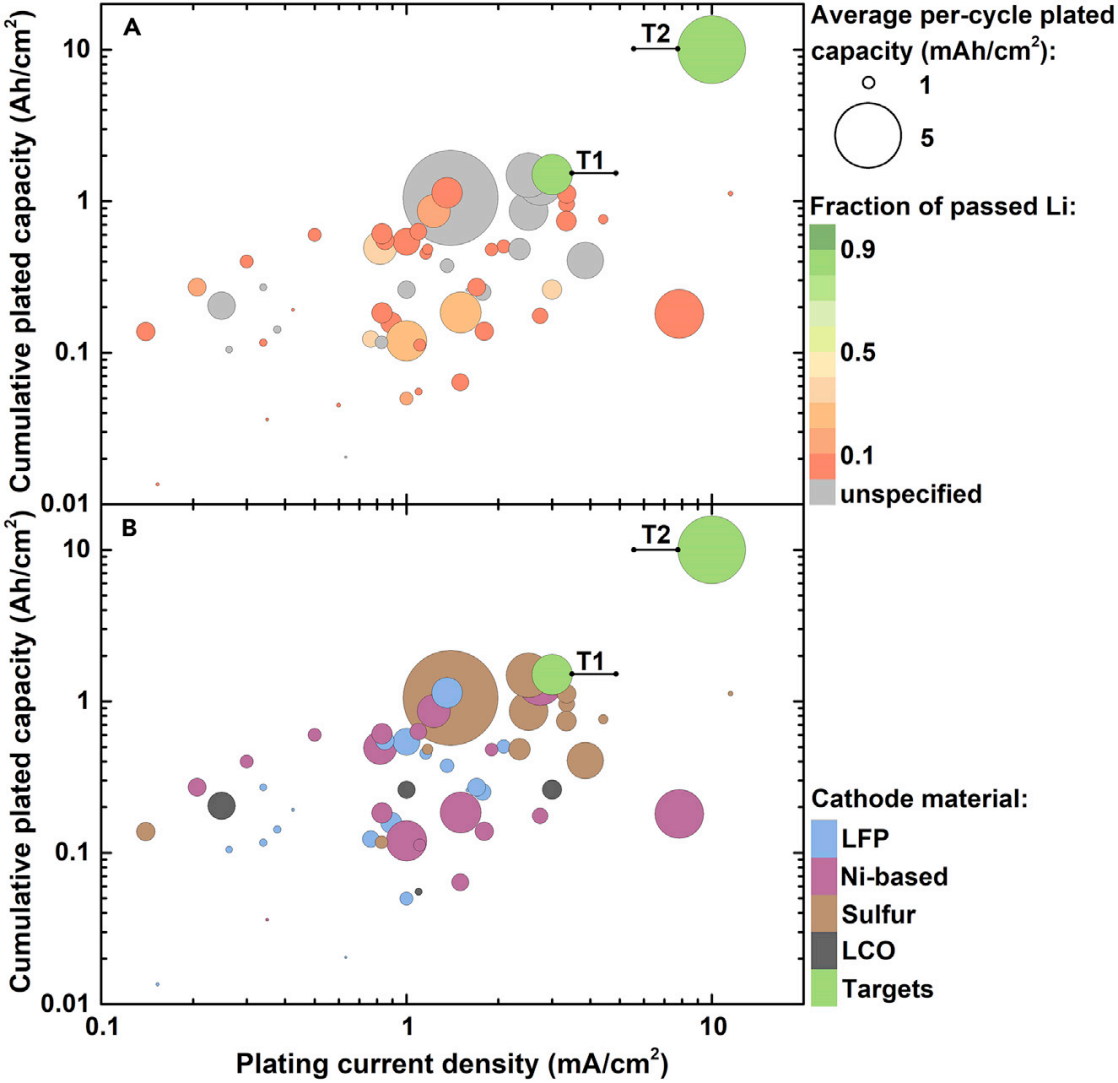
Performance of ASEI-coated anodes in LMBs Performance of ASEI-coated Li metal anodes (or Cu current collectors) in Li metal batteries, analyzed according to 4 parameters: (i) cumulative plated capacity for the reported cycling duration (y axis); (ii) plating current density (x axis); (iii) average per-cycle plated capacity (bubble size); (iv) fraction of initial Li metal passed per cycle (bubble color; gray bubbles correspond to lack of information on the thickness of Li foils) (A); or type of cathode material (bubble color) (B). The ARPA-E IONICS goal (T1) and the fast-charging goal (T2) were included as reference targets. Details on materials and parameters calculations are available Table S2.

The performance of LMBs with ASEI-coated Li metal anodes under full cell test conditions have been evaluated and summarized in Figure 6, based on the following four parameters: (i) plating current density, (ii) cumulative plated capacity, (iii) average per-cycle plated capacity, and (iv) fraction of Li passed per cycle. The cumulative plated capacity was calculated by multiplying the average per-cycle areal capacity for the corresponding number of cycles. The same targets employed for symmetric cells were adopted for evaluating the full cell performance. Details on parameter calculations and reported performance are available in the supplemental information.

The comparison in Figure 6A does not consider the nature of cathode material and the nature and loading of liquid electrolyte. As discussed for symmetric cells, common carbonate- and ether-based electrolytes were used, generally with some additives that could have contributed to improve the cycling stability. A large variety of cathode materials were employed, with LiFePO_4_ (LFP) being the most used, followed by Ni-based cathodes, sulfur, and LiCoO_2_ (LCO). These cathode materials have distinct characteristics, particularly theoretical specific capacity, operating voltage and stability, therefore Figure 6B reports the cycling performance per cathode type (corresponding to different bubbles colors).

LFP is an intercalation cathode material with olivine structure and a theoretical capacity of 170 mAh/g (Nitta et al., 2015). LFP finds application in hybrid EVs and in Chinese passenger EVs, thanks to its high thermal stability, lower cost and improved sustainability compared to other commercial cathodes (Nitta et al., 2015; Sharma and Manthiram, 2020). However, the relatively low average voltage limits the energy density of LFP-based batteries. Nevertheless, LFP remains widely used in academic research on LMBs. In analyzed reports, the typical mass loading of LFP was only a few milligrams, limiting the per-cycle capacity to <1 mAh/cm^2^. This is far from the T1 of 3 mAh/cm^2^, and would require >1500 cycles with high capacity retention to reach a cumulative plated capacity comparable to that of T1. Notably, an average per-cycle capacity of ∼2.3 mAh/cm^2^ for over 500 cycles at 0.5C was reported for a Li|LFP cell with a hybrid ASEI composed of poly(2-chloroethyl acrylate) and Li_3_PS_x_ (Yuan et al., 2019). The good cycling stability was attributed to the simultaneous strength and flexibility of the cross-linked hybrid film, and to the high conductivity of Li_3_PS_x_ and Li sulfides.

LiCoO_2_ (LCO) was the first commercialized cathode for Li-ion batteries, with a high theoretical capacity of 274 mAh/cm^2^ and high discharge voltage (Nitta et al., 2015). The main disadvantages of LCO are its poor thermal stability, the high cost of Co and, more importantly, ethic and health concerns associated with Co extraction (Sharma and Manthiram, 2020). By assembling a pouch cell with 2.24 mAh/cm^2^ LCO and a Li metal anode coated with a thin film (2 μm) of LiPON via sputtering, ∼80% capacity was retained in 100 cycles at 0.1C (Liu et al., 2018).

LiNi_x_Co_y_Mn_z_O_2_ (NCM) are attractive cathode materials with high theoretical capacity (275–280 mAh/cm^2^) and high operating voltage (Nitta et al., 2015). NCM333, NCM523, and NCM622 are increasingly used in the EV market, while the Ni-rich NCM811 will likely be commercialized in the near future (Nitta et al., 2015; Sharma and Manthiram, 2020). These cathodes allows for obtaining specific capacities approaching 200 mAh/g, and even 220 mAh/g for NCM811, thus enabling to design cells with specific energies of >400 Wh/kg (Liu et al., 2019; Chen et al., 2019b). LiNi_0.8_Co_0.15_Al_0.05_O_2_ (NCA) is another cathode material with high theoretical capacity (280 mAh/cm^2^) and increasing application in EVs. NCA cathodes can deliver a practical discharge capacity of 200 mAh/cm^2^; however severe capacity fading is generally observed at a moderate temperature (Nitta et al., 2015). NCM and NCA cathodes with relatively high active material loadings (>10 mg/cm^2^) are increasingly used to test the performance of ASEI-coated Li metal under more realistic conditions. However, the number of cycles with relatively stable capacity is generally <100. Li|NCM333 cell with a reactive PAA (Figure 2) coating on the Li metal anode demonstrated an areal capacity of ∼3 mAh/cm^2^ for 40 cycles (Li et al., 2018a), while Li|NCA cell with a hybrid ASEI composed of YSZ-*g*-PAN achieved a high areal capacity of ∼3.6 mAh/cm^2^ for 50 cycles at 1C (Li et al., 2020a). Longer cycling was observed with the perfluorinated ionomer-coated Li metal anode that paired with a ∼3 mAh/cm^2^ NCA cathode enabled a capacity retention of 94% in 400 cycles at 0.5C (Tu et al., 2017).

Sulfur is a conversion-type cathode material with very high theoretical capacity (1675 mAh/g); the low-cost and abundance of sulfur makes Li-S batteries a highly attractive post Li-ion technology (Nitta et al., 2015). High-energy Li metal batteries such as Li-S and Li-O_2_ enable specific energies at the cell level of >500 Wh/kg (U.S. Department of Energy, 2020; Liu et al., 2019). Several issues hamper the implementation of sulfur cathodes, such as the dissolution of polysulfide intermediates in the electrolyte and dramatic volumetric changes (Nitta et al., 2015). Therefore, ASEIs for Li-S batteries should be designed to simultaneously improve the anode stability and alleviate cathodic problems, for example by blocking polysulfides from reaching Li. Among analyzed reports, Li-S batteries with ASEIs had sulfur loadings <5 mg/cm^2^ and delivered low areal capacities (<2 mAh/cm^2^) for a few hundred cycles, indicating that a combination of anode, cathode, and electrolyte engineering is likely necessary to realize the potential of Li-S cells. A copolymer of poly(3,4-ethylenedioxythiophene) and PEO (PEDOT-*co*-PEG) used as ASEI on the Li anode of a Li-S battery enabled to maintain an average capacity of ∼2.9 mAh/cm^2^ for 300 cycles at 0.5C (Ma et al., 2014a). The cycling stability derived from the ability of PEO to conduct Li^+^ and adhere to the surface of Li metal, blocking the access to polysulfides. Li-S cells with Li-terminated sulfonated TiO_2_ as ASEI and a polyethylenimine-attached rGO (PEIrGO) coating on the cathode side of the separator delivered an average capacity of 3.3 mAh/cm^2^ for 450 cycles at 0.5C. The good performance was attributed to the effective dendrite suppression from the ASEI, combined with the ability of PEIrGO to intercept polysulfides (Kim et al., 2017). Finally, a recent patent reported a high capacity Li-S cell (∼7 mAh/cm^2^) by pairing a Li metal anode coated with 2D materials (e.g. MoS_2_) and an S/carbon nanotubes cathode prepared without a binder to maximize the loading of active material (Choi and Cha, 2020).

### Thin Li anodes and anode-free cells

For practical LMBs, a relatively low loading of Li metal is important to reduce the cell weight, thus increasing the energy density while also decreasing the cost (Liu et al., 2019; Albertus et al., 2018). The ratio of negative to positive electrode areal capacity (N/P) should be below 3. For example, N/P = 3 corresponds to Li anode thickness of ∼50 μm for a cathode with 3 mAh/cm^2^ areal capacity, resulting in a Li utilization of >20%. To further increase the utilization of Li and meet the targets (>80%) while also decreasing costs (Albertus et al., 2018), it would be necessary to further reduce the thickness of Li metal to below 20 μm, approaching N/P = 1.1, which is typical for commercial Li-ion batteries (Liu et al., 2019; Chen et al., 2019b). As discussed for symmetric cycling performance, high excess Li results in very low Li utilization (<10%) and apparent long-term stable cycling. Indeed, the high Li inventory continuously replaces the “dead” Li lost in side reactions during cycling, and negligible capacity fading is observed unless substantial electrolyte depletion occurs (Chen et al., 2019b).

The Li utilization data reported in Figure 6A (bubble colors) indicate that over 80% of publications either employed thick Li metal foils or did not report Li thickness. In a few cases thin commercial Li metal foils (50 μm) were used, or limited amounts of Li (2–9 mAh/cm^2^, corresponding to ∼10–45 μm) were pre-deposited on ASEI-coated Cu current collectors. Some of these works paired the thin Li anodes with small cathode loadings, thus N/P remained >3. The P-doped rGO ASEI was built on 50 μm and 20 μm Li foils used to assemble Li|NMC811 cells with cathode loading of 4.1 mAh/cm^2^ (Kim et al., 2018). The cell with N/P ∼2.5 showed high capacity retention for 350 cycles at 0.3C/0.5C charge/discharge with Li utilization of ∼17%, while the cell with N/P ∼1 showed stable capacity for 200 cycles at 0.2C/0.5C charge/discharge with a higher Li utilization of ∼30%. In a patent describing the use of Sn as ASEI, a Li|NCA cell with N/P ∼3 showed high capacity retention for 110 cycles at 0.5C with ∼21% Li utilization, thanks to the ability of Sn to reversibly alloying with Li (Cho et al., 2019). Cu current collectors coated with an inorganic ASEI composed of Li_3_OCl were pre-treated to deposit ∼30 μm of Li, and then coupled with S cathodes, with N/P of 2.6–2.7 (Han et al., 2020). At high C-rates of 2C and 5C, the cells achieved >80% capacity retention after 1050 and 3000 cycles, respectively, which was mainly attributed to the high conductivity of the ASEI. Li metal with a thickness of ∼10 μm was pre-deposited on Cu current collector coated with PAN fiber array, and subsequently assembled with an LFP cathode of ∼1.5 mAh/cm^2^ loading (Lang et al., 2017). The cell with N/P = 1.3 exhibited a capacity retention of 86% over 100 cycles, with ∼34% Li utilization.

### Liquid electrolyte loading

**Figure 7:**
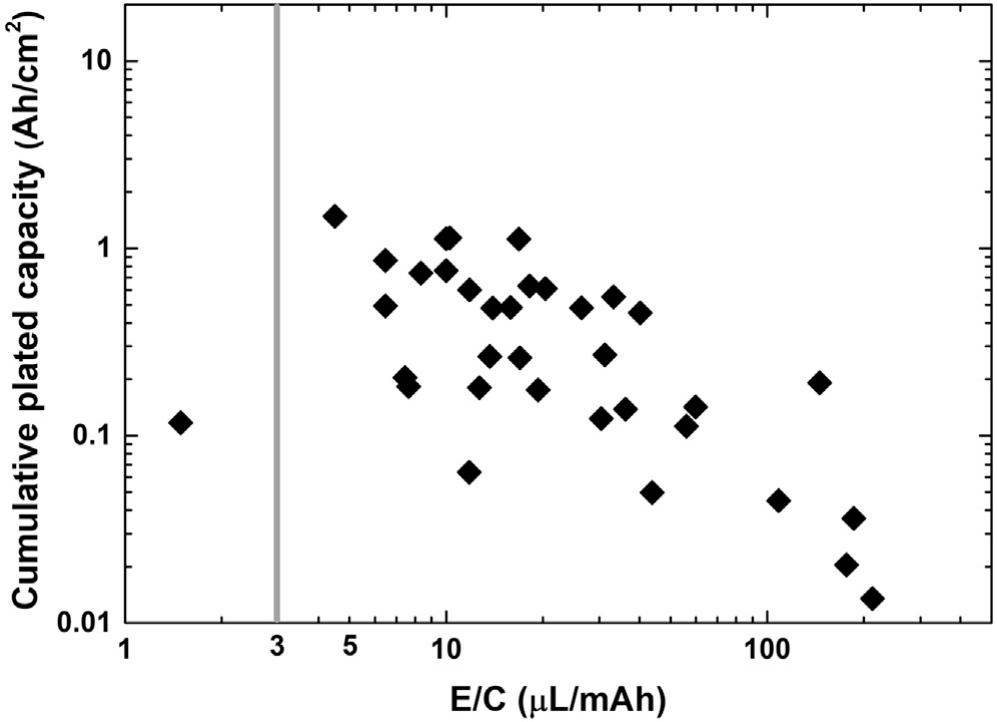
Relation between electrolyte loading to capacity ratio and cell performance Cumulative plated capacity achieved in full cells with ASEI-coated Li metal, as a function of the electrolyte loading to capacity ratio (E/C). The gray line indicates the maximum recommended E/C value (Chen et al., 2019b). When the cathode diameter was not reported (Table S2), the average value of reported diameters (11.76 mm) was used for E/C calculation.

The electrolyte loading contributes to determine the specific energy of practical LMBs, which increases with diminishing the amount of electrolyte. Commercial LIBs have an electrolyte loading of 1.3 g/Ah (or ∼1.3 μL/mAh), expressed as the ratio between the electrolyte amount and the cell capacity (E/C). In a typical LMB, common electrolytes are progressively consumed by reacting with Li metal and therefore lean electrolyte conditions result in rapid cells failure (Fang et al., 2019; Chen et al., 2019b). The full cells analyzed herein were often flooded with electrolyte (E/C >>3 μL/mAh, E/C_average_ ∼42 μL/mAh Figure 7). Under these conditions, eventual electrolyte consumption have marginal effect on the cell performance, thus making difficult to assess the efficacy of the protection provided by the ASEI. When thin Li metal foils and the fraction of plated/stripped Li is significant, a flooded cell can still be stably cycled until nearly all Li is consumed (Chen et al., 2019b). An effective ASEI should prevent the electrolyte consumption, thus enabling lean electrolyte conditions. Therefore, an optimal cell design combines N/P < 3 and E/C < 3 μL/mAh, obtained by using thin Li metal, lean electrolyte, and high cathode loading.

Figure 7 shows the cumulative plated capacity of the full cells with ASEI-coated Li metal as a function of the corresponding E/C value. The electrolyte amount was reported in ∼60% of analyzed works. Only one cell was designed with a very low E/C of ∼1.5. The cell was composed of a sulfur cathode with areal capacity of 2.5 mAh/cm^2^ and a Li metal anode coated by atomic layer deposition (ALD) with a thin layer (8 nm) of ultrapure LiF mixed with LiCO_3_ (Chen, 2019). Under lean electrolyte condition, the cell delivered a stable capacity for 120 cycles at 0.33C.

In addition to employing lean electrolyte conditions, it is important to evaluate the actual electrolyte consumption. This is generally achieved by X-ray photoelectron spectroscopy analysis of the Li electrode surface after cycling, which can reveal the presence of electrolyte decomposition products and their relative amount in comparison to bare Li metal (Li et al., 2016; Chen et al., 2020). Alternatively, nuclear magnetic resonance (NMR) of the electrolyte during/after cycling can be used to quantify the electrolyte consumption. As a notable example, NMR analysis showed that a Li metal anode coated with the P(SF-DOL) polymer (Figure 2) reinforced with GO enabled to retain 77% of a carbonate electrolyte (7 μL/mAh), after 180 cycle (Gao et al., 2019). Conversely, only 41% electrolyte was retained after 50 cycles when using a bare Li metal anode under similar conditions.

## Manufacturing and thickness of ASEIs

### Coating methods

#### Solution-processing methods

Due to their simplicity and relatively low cost, solution-processing techniques are commonly used to build *ex situ* ASEI on Li metal or Cu current collectors. These methods are easily performed inside a glove-box and allows for tuning the thickness and morphology of the ASEI by modifying the solution composition and time of substrate exposure. However, they require relatively high amount of solvents, which can raise the cost and environmental concerns. Solution-processing methods are more often employed for organic and hybrid ASEIs, and include:

i) Drop-casting: consisting in the preparation of a solution containing the active materials, followed by the deposition of a few drops onto the substrate and subsequent solvent evaporation. Surface wetting and rate of evaporation and drying affect the coating properties.
ii) Dip coating or immersion: the substrate is directly immersed in the coating solution, then withdrawn and dried. Surface tension, solution viscosity, immersion time and withdrawal rate of the solution determine the coating characteristics.
iii) Spin coating: the preferred method to obtain highly uniform films with controlled thickness. Upon deposition on the substrate of a solution containing the desired material, the substrate is rotated at a certain rotational speed, which affects the film thickness. Centrifugal forces cause the uniform distribution of the solution.
iv) Doctor blading: consisting of spreading an ink with desired composition across the substrate through a blade placed at a fixed distance from the substrate. After drying, the thickness of the film depends on the blade distance, speed of coating and solution properties. The main advantage is the smaller amount of solvent utilized.
v) Spray coating: a solution with relatively low viscosity is transformed into aerosol by passing through a nozzle. The distance between the nozzle and the substrate is important for the uniformity of the film.

#### Vapor-phase techniques

Vapor phase techniques include chemical and physical deposition methods. They are more commonly used for inorganic ASEIs and enable to obtain uniform coatings with precisely controlled thickness. By using these techniques it is possible to achieve very thin coating layers, in fact inorganic ASEIs typically have thickness <100 nm. This can be beneficial to limit the interfacial resistance and to build a high-energy-density device, as discussed in the next subsection. Conversely, by using solution-processing techniques, it is generally more difficult to finely tune the coating thickness, which typically results in the micron range. However, vapor-phase methods are more demanding in terms of equipment and time than solution-processing methods (Xu et al., 2019).

i) Chemical vapor deposition (CVD) is used to deposit a film on a substrate through chemical reactions with precursors in gas or vapor-phase, and it generally requires a vacuum chamber or a furnace. ALD is a vapor phase technique where the film is grown by building one atomic layer at a time. ALD is increasingly used in the fabrication of batteries, mainly for coating graphite anodes and cathode materials (Kozen et al., 2015; Kazyak et al., 2015). Conformal films of Al_2_O_3_ and LiPON with well-controlled thickness as low as few nanometers were obtained by ALD, which can be performed at low temperature and in inert environment (Kazyak et al., 2015).
ii) Radio-frequency magnetron sputtering uses radio-frequency power to deposit films, predominantly composed of metal oxides or nitrides. It is a type of physical vapor deposition, whereby the coating material is ejected from a target through a sputtering gas. The film thickness depends on the sputtering duration.

#### Other methods

Plasma activation consists of an ultra-fine surface cleaning and modification of the surface topography of the substrate to improve its adhesion property. It can be performed in atmospheric pressure using air or gases, such as N_2_ to create a Li_3_N layer on Li metal (Chen et al., 2019a). The Langmuir-Blodgett film deposition process involves the immersion of the substrate in an aqueous solution containing organic species, which form a homogeneous monolayer over the surface. A modified Langmuir-Blodgett scooping process that exploited the Marangoni effect to form a uniform film at a water/air interface was used to form compact films of rGO and doped rGO, which were then coated on Li metal by a simple roll-press technique (Kim et al., 2018). Other methods include physical pressing of an ASEI on top of Li metal (Yu et al., 2020b) or of a reactive membrane that was removed from the substrate after a pre-determined time (Lang et al., 2019).

### Coating thickness

**Figure 8:**
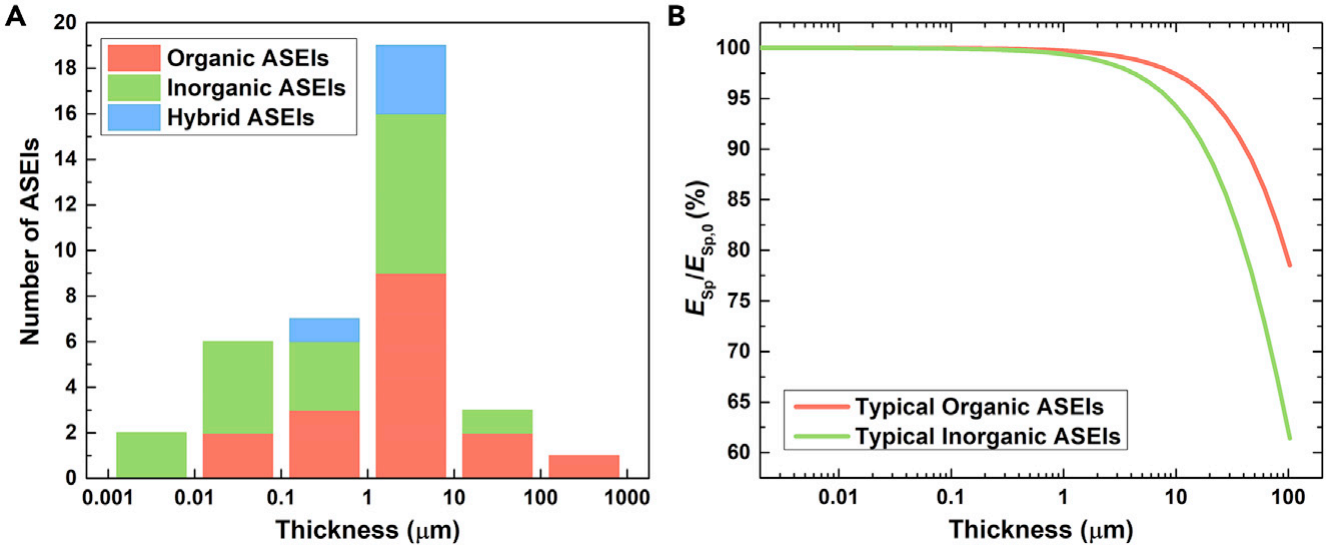
Effect of ASEI thickness on the cell-level specific energy of an LMB (A) Distribution of thickness of ASEIs in the analyzed works that reported this parameter. (B) Estimated percentage decrease in the specific energy *E*_Sp_ of an LMB in a pouch-cell format with optimized cell design to achieve *E*_Sp,0_ = 350 Wh/kg, caused by the introduction of an ASEI on Li metal. The decrease is shown as a function of the ASEI thickness and the nature of the coating material, whereby a density of 1.15 g/cm^3^ is considered for a typical organic ASEI and 2.64 g/cm^3^ for a typical inorganic ASEI. Further details on the calculation are provided in supplemental information.

The thickness of the ASEI affects the cycling performance (Lopez et al., 2018; Kong et al., 2020). Therefore, this parameter is generally optimized by comparing the electrochemical properties and/or the cycling performance of coated Li metal electrodes with variable ASEI thickness, obtained by modifying the coating conditions. If the ASEI is too thick, the Li^+^ transport to the electrode is restricted, the interfacial resistance increases, and the deposition rate slows down (Kong et al., 2020). Conversely, too thin ASEIs may lack uniformity, particularly when solution-based methods are employed, and have insufficient mechanical strength to suppress dendrites (Lopez et al., 2018; Kong et al., 2020). Eventual uncoated spots lead to inhomogeneous Li electrodeposition. Figure 8A shows the distribution of ASEI thickness in the analyzed works that reported this parameter. Only one value per article is reported (i.e. the “optimized” thickness). Thin coatings of tens or hundreds of nanometers are more common for inorganic materials than organics, which instead can have thickness >10 μm and even >100 μm. Regardless the coating nature, the most common thickness range is between 1 and 10 μm.

In a practical LMB, a thick ASEI contributes to the overall cell weight and therefore can cause a non-trivial decrease in the cell specific energy (*E*_Sp_) (Xu et al., 2019). To estimate the effect of the ASEI thickness on *E*_Sp_ of a practical LMB, we considered a model Li|NMC622 pouch-cell composed of 20 layers employing bare Li metal and with optimized electrode and electrolyte loadings to reach a specific energy (*E*_Sp,0_) of 350 Wh/kg (Liu et al., 2019). Figure 8B shows an estimation of the percentage decrease in *E*_Sp_ as a function of the thickness of an ASEI coated on the Li metal in the reference cell. The different trends for organic and inorganic ASEIs depend on the typically lower density of organic materials than inorganic materials (the density of PAA, 1.15 g/cm^3^, was used for organic ASEIs and the density of LiF, 2.64 g/cm^3^, for inorganic ASEIs). ASEIs with thickness <1 μm have negligible impact on *E*_Sp_. However, a coating thickness of 5 μm can decrease *E*_Sp_ of 1.5–3%, while a 10 μm-ASEI can decrease *E*_Sp_ of 2.7–6%. If the thickness further increases to 100 μm, the decrease in *E*_Sp_ can exceed 20%. Despite thicknesses above 10 μm are not common, the possible penalty in *E*_Sp_ when implementing the ASEI in a practical LMB should be considered for optimizing the cell design. At the same time, other parameters can be changed to compensate the reduction in *E*_Sp_. In particular, decreasing the electrolyte amount is likely the most convenient and reasonable step, as the ASEI should avoid electrolyte consumption during cycling. In the case of the reference cell, *E*_Sp,0_ corresponded to E/C = 3 g/Ah (Liu et al., 2019). It can be calculated that decreasing E/C to 2.65 g/Ah can overcome a 3% loss in *E*_Sp_ caused by the ASEI. This estimation further highlights the need for adopting lean electrolyte conditions in full cell tests with coated Li anodes.

## Conclusion and perspectives

Coating Li metal anodes with an ASEI can minimize the loss of Li and electrolyte, and suppress dendrites growth in LMBs, accelerating the commercialization of safe, high-energy LMBs with long cycle life. Functional groups and structural features of reported ASEIs have specific roles in regulating the transport of Li ions and/or in mechanically suppressing the growth of dendrites. The performance comparison reported herein revealed that by incorporating various materials and functionalities it is possible to combine their advantages and prolong the cycling stability of Li metal electrodes. This is facilitated by recent progress in polymers and hybrid materials synthesis that allowed for preparing single-ion conducting coatings, uniform and mechanically strong films of nanoparticles grafted with polymer chains, and conformal and self-healing films based on dynamic networks.

Taking actual targets for high-energy batteries as reference, our analysis highlights that it is necessary to adopt practical testing conditions to transfer the ASEI concept into commercial applications. Tests should be conducted at current densities ≥3 mA/cm^2^ and areal capacities ≥3 mAh/cm^2^, with thin Li metal electrodes (≤20 μm) in symmetric cell tests, and N/P ≥ 3, E/C ≤ 3 μL/mAh in full cells. Cathode materials with high specific capacities should be preferred, particularly Ni-rich cathodes, as it becomes imperative to minimize or even eliminate Co. In addition, the testing conditions must be fully specified in publications, including the thickness of Li metal, the loading and nature of electrolytes and additives, the loading of active material in the cathode and electrodes structural features (e.g. size, thickness, porosity).

Finally, the cost of ASEI materials and manufacturing should also be considered, and a techno-economic assessment of Li metal coatings could complement this review. Several materials such as GO and commodity polymers, and solution-processing techniques have low cost, and therefore appear more suitable for large-scale production of ASEI-coated electrodes.

## Limitations of the study

This review considers ASEIs designed for Li metal batteries operating with liquid electrolytes, therefore examples of ASEI-coated Li metal electrodes coupled with inorganic or polymer solid electrolytes were not included in the performance analysis. Representative ASEI materials were selected, thus if multiple reports were available on a similar material only one of them was considered. The comparative performance shown herein depends on the employed testing conditions, therefore it is not impossible that some materials will provide good performance under different, more harsh testing conditions. Finally, the last part of the review briefly discusses the manufacturing of ASEIs representing a useful starting point for a techno-economic assessment on this technology.

## Methods

All methods can be found in the accompanying transparent methods supplemental file.

## Supplemental information

Document S1. Transparent methods

## Footnotes

Supplemental information can be found online at https://doi.org/10.1016/j.isci.2021.102578.
