# Supplementary material for: Comparative performance of ex situ artificial solid electrolyte interphases for Li metal batteries with liquid electrolytes
Source: iScience. 2021 May 21;24(6):102578. doi: 10.1016/j.isci.2021.102578 (PMC8184660; doi:10.1016/j.isci.2021.102578)
Supplement: Document S1. Transparent methods [file mmc1.pdf]

iScience, Volume *24*

Supplemental information

Comparative performance of ex situ artificial solid electrolyte interphases for Li metal batteries with liquid electrolytes

Francesca Lorandi, Tong Liu, Marco Fantin, Joe Manser, Ahmed Al-Obeidi, Michael Zimmerman, Krzysztof Matyjaszewski, and Jay F. Whitacre

Supporting Information

Calculation details for performance of ASEI-coated Li metal or Cu current collectors in Li|Li and Li|Cu cells (Figure 3, Figure 4 and Figure 5 in main text, and Table S1)

Cumulative plated capacity (Ah/cm^2) = Per-cycle plating capacity (mAh/cm^2)/cycle number/1000

Li fraction (total Li) = (Per-cycle plating capacity (mAh/cm^2)*4.948 (μm*cm^2/mAh))/(2*Thickness_Li_metal(μm))

in case of ASEI@Cu:

Li fraction (total Li) = (Per-cycle plating capacity (mAh/cm^2)*4.948 (μm*cm^2/mAh))/(Thickness_pre-deposited_Li_metal(μm) + Thickness_Li_metal(μm))

where thickness_pre-deposited_Li_metal(μm) = 0 if no Li was pre-deposited underneath the ASEI on Cu current collector (Li|Cu half cells)

Calculation details for performance of ASEI-coated Li metal or Cu current collectors in full cells (Figure 6, and Figure 7 in main text, and Table S2).

Cumulative plated capacity (Ah/cm^2) = Average per-cycle plating capacity (mAh/cm^2)/cycle number/1000 = ((Initial per-cycle plating capacity + final per-cycle plating capacity)/2 (mAh/cm^2))/cycle number/1000

Li fraction (total Li) = (Per-cycle plating capacity (mAh/cm^2)*4.948 (mm*cm^2/mAh))/(Thickness_Li_metal(μm) + cathode capacity (mAh/cm^2)*4.948 (μm*cm^2/mAh))

except for non-lithiated cathode materials (e.g. S):

Li fraction (total Li) = (Per-cycle plating capacity (mAh/cm^2)*4.948 (mm*cm^2/mAh))/(Thickness_Li_metal(μm))

in case of anode-free batteries: Thickness_Li_metal(μm) = 0

Theoretical capacity of cathode materials:

unless otherwise stated in the paper/patent, the following theoretical specific capacity of different cathodes materials were used for calculations:

LFP: 170 mAh/cm^2

LCO: 274 mAh/cm^2

NCMs and NCA: 275 mAh/cm^2

S: 1675 mAh/cm^2

Details on calculation of ASEI thickness effect on cell specific energy (Figure 8)

LMB Pouch cell characteristics (Liu et al., 2019)

Size: 70.0x41.5x4.5 mm

Layers: 20

Cathode material: NCM622

Cathode loading: 22 mg/cm^2

Specific capacity: 196 mAh/g (charged at 4.6 V)

Average discharge voltage: 3.89 V

N/P: 2.4

E/C: 3 g/Ah

Total weigth: 0.0252 kg

→ $E_{Sp,0}$ = 350 Wh/kg

ASEI characteristics

Area: 70.0x41.5 mm

Density (organic): 1.15 g/cm^3 (Sigma Aldrich)

Density (inorganic): 2.64 g/cm^3 (Wikipedia)

References:

Liu, J., Bao, Z., Cui, Y., Dufek, E. J., Goodenough, J. B., Khalifah, P., Li, Q., Liaw, B. Y., Liu, P., Manthiram, A., Meng, Y. S., Subramanian, V. R., Toney, M. F., Viswanathan, V. V., Whittingham, M. S., Xiao, J., Xu, W., Yang, J., Yang, X.-Q. Zhang, J.-G. (2019). Pathways for practical high-energy long-cycling lithium metal batteries. Nat. Energy *4*, 180-18.

Sigma Aldrich (density of commercial poly(acrylic acid)) [Online]. Available: https://www.sigmaaldrich.com/catalog/product/aldrich/416002?lang=en&region=US [Accessed March 2021]

Wikipedia (density of LiF salt) [Online]. Available: https://en.wikipedia.org/wiki/Lithium_fluoride [Accessed March 2021]
